# Isolation and Characterization of Halotolerant Plant Growth Promoting Rhizobacteria From Durum Wheat (*Triticum turgidum* subsp. *durum*) Cultivated in Saline Areas of the Dead Sea Region

**DOI:** 10.3389/fmicb.2019.01639

**Published:** 2019-07-23

**Authors:** Randa N. Albdaiwi, Hala Khyami-Horani, Jamal Y. Ayad, Kholoud M. Alananbeh, Rabea Al-Sayaydeh

**Affiliations:** ^1^Department of Biological Sciences, Faculty of Science, The University of Jordan, Amman, Jordan; ^2^Department of Horticulture and Crop Science, Faculty of Agriculture, The University of Jordan, Amman, Jordan; ^3^Department of Plant Protection, Faculty of Agriculture, The University of Jordan, Amman, Jordan; ^4^Shoubak College, Al-Balqa‘ Applied University, Shoubak, Jordan

**Keywords:** ACC deaminase, durum wheat, endophyte, halotolerant PGPR, salinity

## Abstract

Plant growth promoting rhizobacteria (PGPR) are beneficial microorganisms that can be utilized to improve plant responses against biotic and abiotic stresses. In this study, 74 halotolerant bacterial isolates were isolated from rhizosphere and endorhizosphere of durum wheat (*Triticum turgidum* subsp. *durum*) plants cultivated in saline environments in the Ghor region near the east of the Dead Sea. *16S rDNA* partial sequences and phylogenetic analysis of 62 isolates showed clear clustering of the isolates into three phyla: Firmicutes (61.3%), Proteobacteria (29.0%), and Actinobacteria (9.7%). At the genus level, the majority of them were grouped within the *Bacillus, Oceanobacillus*, and *Halomonas* genera. The isolates, which possessed plant growth promoting traits including nitrogen fixation, ACC deaminase activity, auxin production, inorganic phosphate solubilization and siderophore production, were selected. The effect of the inoculation of selected PGPR strains on growth of salt sensitive and salt tolerant durum wheat genotypes under high salt stress conditions was evaluated. Six halotolerant PGPR strains were able to improve survival in inoculated plants under high salinity stress conditions as reflected in higher germination percentages and seedling root growth when compared with non-inoculated plants. Furthermore, three halotolerant PGPR strains were able to improve durum wheat tolerance to water deficit stress. In addition, antagonistic effect in four halotolerant PGPR strains against an aggressive pathogenic isolate of *Fusarium culmorum* that causes crown rot disease was observed in a dual culture assay. In conclusion, the halotolerant PGPR strains described in this study might have great potential to improve durum wheat productivity under different stress conditions.

## Introduction

Agricultural productivity is severely affected by major biotic and abiotic factors including drought, salinity, extreme temperatures and pathogens, which can limit the growth and development of any given crop. Salinity is an adverse condition affecting crop productivity in arid and semi-arid areas around the world where it causes an annual loss of 1–2% of arable land (Shrivastava and Kumar, 2015). Salinity alters cellular metabolism causing many physiological, morphological, biochemical, and molecular changes in plants (Gupta and Huang, 2014). Salinity impact on plant growth and development is primarily due to imposing osmotic stress, which has an immediate effect on water availability, accumulation of toxic ions such as Na^+^ and Cl^–^ in the cells, nutrient imbalances, and oxidative stress damage (Munns and Tester, 2008).

Wheat (*Triticum* spp.) is considered one of the most important crops in the world and it is a staple food for over 35% of the world’s population where it provides more calories and proteins than any other cultivated crop (FAOSTAT, 2017). Durum wheat (*Triticum turgidum* subsp. *durum*) is grown on 10% of all wheat cultivated areas in the world, and it is a major cereal crop in the Mediterranean region. Several studies indicated that the wild progenitor of modern durum wheat is widely distributed in the Jordan Valley region nearby the Dead Sea (Nevo et al., 2013) with archeological evidences of durum wheat utilization near the Dead Sea region before 9500 years ago (Weide, 2015).

High salt stress has more pronounced effects on durum wheat growth and development when compared with other cereals (Munns et al., 2006). This is mainly due to its inability to exclude Na^+^ from its tissues (Roy et al., 2014). Several approaches were used to reduce salinity effects on durum wheat, which included proper soil practices and irrigation managements (Katerji et al., 2009) as well as traditional breeding and genetic engineering (Munns et al., 2006).

Microorganisms associated with the rhizosphere play a significant role in alleviating salt stress in plants resulting in better crop productivity (Etesami and Beattie, 2018). Among these, bacteria known as plant growth promoting rhizobacteria (PGPR), may possess multiple plant growth promoting traits able to increase plant growth and yield of crops and useful in directly and indirectly alleviating the effect of abiotic stresses. Directly, PGPR facilitate plant nutrients uptake from surrounding environments by producing siderophores to sequester iron and/or by phosphorus solubilization and/or by nitrogen fixation (Etesami and Beattie, 2018). Furthermore, PGPR can modulate plant growth by providing phytohormones such as indole acetic acid (IAA) or reducing the ethylene production by the activity of the 1-Aminocyclopropane-1-carboxylate (ACC) deaminase enzyme (Glick, 2014). On the other hand, indirect plant growth promotion by PGPR occurs when they limit or prevent plant damage caused by pathogenic agents such as bacteria, fungi and nematodes (Compant et al., 2005). Several studies have been conducted in order to understand the role of halotolerant PGPR on alleviating salinity damages on wheat plant (reviewed in Numan et al., 2018), with few reports on durum wheat. For instance, the inoculation of *Waha* durum wheat cultivar with *Azospirillum brasilense* NH strain, isolated from a saline soil in northern Algeria, improved the growth under salt stress conditions (Nabti et al., 2010).

The main objective of this study is to isolate halotolerant PGP rhizospheric and endophytic bacteria associated with durum wheat grown in saline manifested soils near the eastern side of the Dead Sea. The isolates were partially identified by *16S* rDNA sequencing and tested *in vitro* for their abilities to promote plant growth. Selected halotolerant PGPR isolates were evaluated for growth promoting ability in durum wheat plants grown under different levels of salinity.

## Materials and Methods

### Collection Sites

During February and March 2017, soil and plant samples were collected from fields cultivated with durum wheat cv. *Haurani*, from three sites near the northern and eastern sides of the Dead Sea (−430 m, latitude: 31.3333° N, longitude: 35.5000° E): Ghor Haditha, Ghor Safi, and Ghor Sweimeh (Supplementary Table S1). These regions are characterized by an arid and very warm Mediterranean climate with a mean annual precipitation less than 100 mm and maximum temperatures above 40°C during summer (AL-Zu’bi and Al-Kharabsheh, 2003). From each field, three soil and three plant samples were collected in sterile plastic bags and placed in ice packs before their transfer to the laboratory of Plant Biotechnology, Faculty of Agriculture, The University of Jordan. Collected soil samples were analyzed for chemical and physical properties (Supplementary Table S1): the soil texture was analyzed by the Hydrometer method, organic matter by the Walkley-Black method and Electrical Conductivity (EC) and pH by saturated paste extract method (Estefan et al., 2014).

### Isolation of Rhizospheric and Endophytic Bacteria

To isolate rhizospheric bacteria, plant roots were gently shaken to remove the clumps of loosely adhering soil to the roots, leaving behind the root-firmly adhering soil particles (rhizospheric soil), which was then suspended and vortexed in 100 ml of sterile 1% NaCl solution. Thereafter, 10 folded serial dilutions were prepared and 100 μl from each diluent were plated on nutrient agar (NA) medium (Thermo Fisher Scientific Oxoid, Ltd., Basingstoke, United Kingdom) supplemented with 10% NaCl. The plates were incubated at 28°C and monitored for colony formation up to 1 week (Fischer et al., 2007).

For the isolation of endophytic bacteria, the roots of the collected samples were washed carefully under running tap water for 10 min to remove adhering soil particles. The roots were disinfected with 70% ethanol for 1 min, then rinsed three times with sterile distilled water. The roots were then surface sterilized with 3% sodium hypochlorite solution containing few drops of Tween 20^®^ (Sigma-Aldrich, Steinheim, Germany) for 10 min followed by six rinses with sterile distilled water. To confirm root surface sterilization efficiency, an aliquot (100 μl) from the sixth wash solution was spread on NA supplemented with 10% NaCl and incubated at 28°C for 5 days. Thereafter, 1 g of the surface sterilized root tissue was macerated with a sterilized mortar and pestle in 10 ml of 1% NaCl solution, and serial dilutions were prepared and 1 ml from the tissue extract and the diluents were spread on NA medium supplemented with 10% NaCl. The plates were incubated at 28°C and monitored up to 1 week for bacterial colony formation (Ramadoss et al., 2013).

The colonies of rhizospheric and endophytic bacterial isolates were examined morphologically for their shape, size, margin, elevation, appearance, texture, pigmentation, and optical properties. In addition, cellular morphology, shape, gram staining and endospore formation were also examined (Robinson et al., 2016) (Supplementary Table S2). Colonies with distinct morphological characteristics were selected and purified by subculturing three times on NA media supplemented with 10% NaCl, before their storage in a 40% glycerol solution at −80°C till further use. Each sample was given a code representing the collection site (GHD: Ghor Haditha, GSF: Ghor Safi, and GSW: Ghor Sweimeh), followed by either R for Rhizospheric or E for Endophytic and by the isolate number (Supplementary Table S2).

### Halotolerance Assay

Bacterial isolates were screened for halotolerance using NA media supplemented with various levels of NaCl (1, 5, 10, 15, 20, and 25%). The plates were inoculated with fixed volumes of starter inoculum (OD_600_ = 0.05) and the cultures were incubated for 7 days at 28°C (Ramadoss et al., 2013).

### *16S rDNA* Sequencing and Phylogenetic Analysis

The selected bacterial isolates were partially identified by *16S rDNA* sequencing and phylogenetic analysis. For this purpose, 5 ml cultures grown in liquid NA media were used for total genomic DNA isolation using the Wizard^®^ Genomic DNA Purification Kit (Promega, Madison, WI, United States) following the manufacturer’s instruction. The isolated gDNA from each isolate was assessed by gel electrophoresis in a 1% agarose gel stained with Red Safe (Intron, BioTek, South Korea) and visualized by using Gel Doc^TM^ XR Gel Documentation System (Bio-Rad, Hercules, CA, United States). The DNA concentration and quality were determined by spectrophotometry (Smart-Spec^TM^ plus spectrophotometer, Bio-Rad, Hercules, CA, United States) and a stock solution (30 ng/μl) for each isolate was prepared and stored at −20°C for further use.

For the amplification of the *16S rDNA* region, PCR assays were performed by using the *16S rDNA* bacterial universal primers: Forward (5′-AGAGTTTGATCCTGGCTCAG-3′) and Reverse (5′-AAGGAGGTGATCCAGCCGCA-3′) (Edwards et al., 1989). The PCR reaction was performed in a 25 μl reaction mixture containing 30 ng of gDNA as a template, 2.5 μl of primers mix (10 μM of each primer), 5 μl of (5X) PCR buffer, 1.5 μl of (25 mM) MgCl_2_, 0.5 μl of (10 mM) dNTPs, 0.5 μl of (50 unit/ μl) *Taq* polymerase (Promega, Madison, WI, United States) and the final volume was brought to 25 μl by using nuclease free water. PCR was carried out by using thermal cycler (BIO-RAD C1000^TM^ Thermal Cycler, United States) with the following amplification conditions: 94°C for five min for initial DNA denaturation, 30 cycles at 94°C for 1 min (denaturation), 57°C for one min (annealing) and 72°C for 1.5 min (extension), and a final elongation step at 72°C for five min. The amplified products were analyzed by gel electrophoresis as described earlier and were purified by Wizard^®^ SV Gel and PCR Clean-Up System (Promega, Madison, WI, United States), following the manufacturer’s instructions. The final purified PCR products were sequenced from both directions using the *16S rDNA* Forward and Reverse primers at Macrogene, Inc., (Seoul, South Korea) using an ABI 3730XL capillary electrophoresis sequencing station (Applied Biosystem, United States).

The *16S rDNA* sequences of the bacterial isolates were compared against sequences available in the GenBank by the BLASTn tool^1^ using non-redundant (nr) and microbes databases. The phylogenetic analysis of the *16S rDNA* sequences of bacterial isolates with reference bacterial sequences identified in the BLAST search were carried out using the MEGA6.0 software package^2^ (Tamura et al., 2013). The sequences were aligned using the embedded Muscle algorithm and the output was used to build a phylogenetic tree by calculating distance matrices for neighbor joining (NJ) analysis with the Kimura two-parameter model and a bootstrapping analysis with 10000 replicates to test the robustness of internal branches. Several *16S rDNA* sequences of previously identified PGPR were included as references in the phylogenetic tree.

### *In vitro* Assessment of Plant Growth Promoting Traits

Plant growth promoting traits of bacteria isolates were assessed for nitrogen fixation, inorganic phosphate solubilization, siderophore production, IAA production, ACC deaminase activity, and antifungal activity against *Fusarium culmorum*. A reference PGPR strain, *Azospirillum lipoferum* (ATCC^®^ 29707^TM^), was used as positive control in all assays except for ACC deaminase assay as it served as a negative control. All assays were carried out in triplicates and were replicated at least three times for each assay.

#### Nitrogen Fixation Assay and PCR Amplification of the *nifH* Gene

The qualitative estimation of nitrogen fixation was conducted as described by Baldani et al. (1986). The nitrogen free semi-solid (NFb) medium was prepared and inoculated with fixed volumes of a starter inoculum (OD_600_ = 0.05) and the cultures were then incubated at 30°C for 5 days and monitored for the formation of a pellicle at the subsurface level. Blue color development was considered a positive sign for nitrogen fixation. To confirm the positive results, the bacteria were inoculated on NFb solid medium [supplemented with 10 g Agar (Thermo Fisher Scientific Oxoid, Ltd., Basingstoke, United Kingdom)] at 28°C for 7 days and bacterial growth and the formation of blue color were used as qualitative evidences for nitrogen fixation.

Further confirmation of nitrogen fixation ability was carried out by PCR amplification of a targeted fragment within the *nitrogenase iron protein* (*nifH*) gene. For this purpose, all isolates that gave positive results in the qualitative assay and selected negative isolates were tested for *nifH* genes (Ji et al., 2014). For this purpose, a *nifH* gene fragment (∼390 bp) was amplified by using two universal primers: 19F (5′-GCIWTYTAYGGIAAR GGIGG-3′) and 407R (5′-AAICCRCCRCAIACIACRTC-3′). The amplified products were analyzed by gel electrophoresis were carried out as previously described.

#### Phosphate Solubilization Assay

The ability of inorganic phosphate solubilization was conducted by spot inoculation of bacterial isolates on modified Pikovskaya agar plates using tricalcium phosphate as a substrate (Goswami et al., 2014). The formation of transparent halo zones around the bacterial colonies after 7 days of incubation at 28°C were considered an indication of phosphate solubilizing activity.

#### Siderophores Production Assay

The siderophore production was assayed by spot inoculation of selected bacterial isolates on chrome azurol S (CAS) blue agar plates as described by Schwyn and Neilands (1987). The cultures were incubated for 7 days at 28°C on CAS blue agar plates. The formation of halo zones around the growing colonies was monitored and bacterial isolates with clear zones were scored as siderophore producers.

#### Indole Acetic Acid Production Assay

Indole acetic acid production by selected bacterial isolates was determined using a modified quantification method developed by Bric et al. (1991). Briefly, the bacterial isolates were cultured for 24 h in 10 ml of NB and then, 100 μl of bacteria inoculum (OD_600_ = 0.5) for each selected isolate was transferred into 5 ml of NB medium supplemented with 1% of L-tryptophan (Sigma-Aldrich, Germany). After the incubation in the dark conditions at 30°C with continuous shaking (150 rpm) for 4 days, 2 ml of the bacterial cultures were centrifuged (8,000 rpm and 4°C) and 70 μL of the supernatant were transferred into a well in a microtitre plate. Thereafter, 140 μl of Salkowski reagent (50 ml of 35% HClO_4_ +1 ml of 0.5M FeCl_3_) was added to the supernatant and the mixture was kept at room temperature for 25 min. The absorbance at 530 nm was measured using an Epoch plate reader (BioTek, United States) (Tsavkelova et al., 2007). The un-inoculated tryptophan containing medium mixed with the Salkowski reagent was used as a negative control. The development of pink color in the well-indicated the production of IAA, and the amount of IAA produced was estimated against a standard curve prepared with different concentrations of IAA (Sigma-Aldrich, Germany).

#### ACC Deaminase Assay and PCR Amplification of the *acdS* Gene

The ACC deaminase activity of bacterial isolates was assessed based on the ability of the respective isolate to use ACC as a sole nitrogen source in a nitrogen free broth (Glick et al., 1995). Selected bacterial isolates were grown in 10 ml of NB medium at 28°C for 24 h. The cultures were then centrifuged for five min at 8,000 rpm and the pellets were washed twice in one ml of normal saline before spot inoculation on Burks’ media supplemented with three mM ACC (Sigma-Aldrich, United States) as the sole nitrogen source. ACC-free Burks’ media with and without 0.2% Ammonium Sulfate ((NH_4_)_2_SO_4_) were used as positive and negative control, respectively. The cultures were incubated for 7 days at 28°C and the growth of bacterial isolates on ACC-supplemented plates were compared with the negative and positive controls (Ali et al., 2014).

To confirm the ACC deaminase assay results, PCR amplification of an *ACC deaminase* (*acdS*) gene fragment (∼800 bp) was conducted in positive and negative isolates using gene specific primers as described by Jha et al. (2012). For this purpose, *acdS* gene was amplified by using two universal primers: Forward (5′-GCCAARCGBGAVGACTGCAA-3′) and Reverse (5′-TGCATSGAYTTGCCYTC-3′). The amplified products were analyzed by gel electrophoresis and visualized by using Gel Documentation System as previously described.

#### Antifungal Activity Against *F. culmorum*

Antifungal activity of nine selected bacterial isolates against a *F. culmorum* aggressive isolate (accession number MH001550.1), which causes crown rot disease on wheat in Jordan (Alananbeh, unpublished), was tested using a dual culture method (Ji et al., 2014). The inoculated plates were incubated at 28°C for 10 days and the antagonistic effects of the isolates against an *F. culmorum* isolate were monitored for the formation of inhibition zones starting from day 3. The dual culture experiments were repeated three times.

### Evaluation of Growth Promoting Ability in Durum Wheat

#### Salinity Experiments

Based on *in vitro* plant growth promoting assays, nine isolates possessing most of plant growth-promoting traits were selected and used to evaluate their effect on two durum wheat genotypes at the germination stage in the presence of different salinity levels. Two durum genotypes were selected: Tamaroi, a salt sensitive Australian cultivar, and Line 5004, a salt tolerant BC4F2 homozygous line carrying *NAX2* (*TmHKT1;5-A)* gene derived from backcrossing line 149 with Tamaroi (recurrent parent) (kindly provided by Dr. S. Udupa, ICARDA, Rabat, Morocco; James et al., 2006).

The seeds of the two lines were surface sterilized by washing with 70% ethanol for 1 min followed by three washings with sterile distilled water., Thereafter, the seeds were treated with 1.5% sodium hypochlorite (NaOCl) solution for 5 min followed by six times of successive washings in sterile water to remove all traces of the disinfectant (Rudolph et al., 2015). To check the efficacy of sterilization process, few seeds were placed on plates containing NA medium for 4 days and the plates were monitored for any microbial growth.

To prepare the bacteria inoculum, log phase cultures (OD_600_ of ∼0.6) of the nine selected bacterial isolates and *A. lipoferum* (ATCC^®^ 29707^TM^) were used. The cultures were centrifuged at 5000 rpm for 10 min and the pellets were washed three times with sterile distilled water and then resuspended in a final volume of 25 ml sterile distilled water. Sterilized seeds were imbibed in the bacterial suspension for 1 h (for control treatment, seeds were imbibed in sterile water). Imbibed seeds were allowed to dry under laminar air flow cabinet for 2 h before transferring them into sterile plastic boxes lined with sterile filter papers soaked with 30 ml of different concentrations of NaCl (0, 80, 120, 160, and 200 mM). Three replicates for each treatment was performed with 20 seeds per plate (10 seeds of Tamaroi and 10 seeds of Line 5004) and the experiments were repeated twice. All boxes were incubated at 22°C under complete darkness for the first 3 days, then placed under a photoperiod cycle of 14 h light–10 h dark for other 7 days. Germination percentage was measured after 10 days and root length and seedling dry weight were recorded.

The same germination experiment was repeated twice with three selected bacterial isolates (out of nine) showing clear growth promoting activity and a negative control (non-inoculated seeds) using different concentrations of NaCl (80 and 160 mM of NaCl) and non-saline conditions to evaluate their effect on root projected area, root length, and root diameter using the WinRhizo root scanning software system (version 2009c; Regent Instruments, Inc., Quebec City, QC, Canada).

The effect of plant growth promoting activity of a selected isolate from wheat (GSW-E-7) on seedling growth under saline conditions was carried out using a hydroponic culture. For this purpose, sterilized seeds of the two durum genotypes (Tamaroi and Line 5004) were inoculated with GSW-E-7 bacterial strain, in addition to non-inoculated as a control. The seeds of both treatments germinated on filter paper wetted with distilled water as previously described above. Thereafter, 1-week old seedlings were transferred into a sterilized aerated hydroponic system that consisted of a dark plastic box (capacity 10 L) filled with a nutrient solution (Hoagland and Arnon, 1950) supplemented with different NaCl concentrations (0, 80, and 160 mM). Each box was aerated with an adjustable air pump that was operating continuously to ensure adequate air supply. In each box, six seedlings from each genotype inoculated with the bacterial isolate were grown for a period of 3 weeks in a controlled growth chamber under a daily temperature regime of 24 ± 1°C/ day and 18 ± 1°C/ night with a photoperiod of 12 h light and 12 h darkness. Each treatment was replicated three times and the experiments were repeated twice. At the end of each experiment, data were collected for shoot and root fresh weights, projected areas and lengths for shoots and roots.

#### Drought Experiments

The nine selected bacterial isolates described above were used to assess their effect on drought tolerance in 2-week-old of durum wheat cv. *Tamaroi*. For this purpose, 5 sterilized seeds inoculated with a selected bacterial isolate and non-inoculated seeds (negative control) were sown in a small pot (10 cm depth and 10 cm diameter) filled with sterile sand. Three replicates for each treatment were used. After seedling emergence, pots were kept moist for 2 weeks by irrigating them with nutrient solution (Hoagland and Arnon, 1950). Thereafter, drought conditions were imposed by continuous water withholding for 10 days followed by re-watering as described in Al-Abdallat et al. (2014). The wilting behavior of treated seedlings and the survival percentages of inoculated and the non-inoculated control were monitored and each bacteria strain was compared with its negative control.

### Statistical Analysis

The data for salinity experiments were analyzed as a split-plot arrangement of treatments combination in a randomized complete block design. Combined analyses of variance (ANOVA) were performed for bacterial isolates, salinity level and genotypes and their possible combinations for each experiment using Statistical Analysis System (SAS) software (SAS Institute 2002, SAS/Stat software, Release 9.0., Cary, NC, United States) using mixed procedure and means for bacteria × genotype at the same salinity level were separated using Fisher’s protected least significant difference (LSD) at probability level (*p* ≤ 0.05).

## Results

### Collection Sites

In this study, saline areas near the eastern side of the Dead Sea cultivated with durum wheat were surveyed for the isolation of rhizospheric and endophytic bacteria (Supplementary Table S1). The surveyed wheat plants varied in their growth and developmental stages. Ghor Haditha and Ghor Sweimeh plants were at the heading stage, while at Ghor Safi they were at the tillering stage. The plant material collected in Ghor Safi were suffering from environmental stresses and particularly salinity, thus only plants surviving and showing good growth were collected.

The soil texture in Ghor Haditha and Ghor Safi was sandy loam while in Ghor Sweimeh it was sandy-clay-loam. Soil pH ranged from 7.83 to 8.10 and the highest soil organic matter content (6.22%) was recorded in Ghor Safi location (Supplementary Table S1). For soil salinity level, the EC values varied and was 3.32 dS/m in Ghor Haditha, 6.64 dS/m in Ghor Sweimeh and in Ghor Safi it reached up to 17.7 dS/m, which is considered very high indicating a severe saline condition that had negative effect on plant growth and development (Supplementary Table S1).

### Isolation of Rhizospheric and Endophytic Bacteria

Out of 74 isolates that grew on NA media supplemented with 10% NaCl (38 endophytic and 36 rhizospheric), 20 were isolated from Ghor Haditha (13 endophytic and 7 rhizospheric), 32 from Ghor Safi (17 endophytic and 15 rhizospheric), and 22 from Ghor Sweimeh (8 endophytic and 14 rhizospheric) (Supplementary Table S2). The majority of isolated colonies had creamy colored (44.5%), and 62.2% were Gram positive, and rod shaped and most of them were sporeformers. On the other hand, 37.8% of isolates were Gram negative with the majority of them have rod shape. 71.0% of endophytic bacteria and 52.7% of rhizospheric bacteria were Gram positive (Supplementary Table S2). For halotolerance assay, thirty isolates were able to tolerate salinity levels up to 20% NaCl and only seven isolates tolerated up to 25%, six of which were isolated from Ghor Swiemeh (Supplementary Table S3).

### *16S rDNA* Sequencing and Phylogenetic Analysis

**FIGURE 1:**
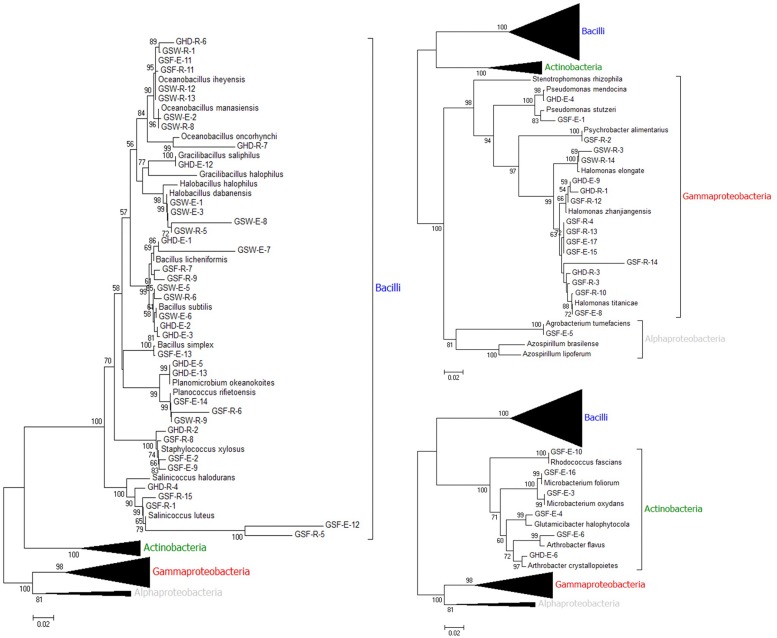
Phylogenetic tree constructed by using neighbor-joining analysis between 62 bacterial isolates [endophytic (E) and rhizospheric (R) and grouped based on class] collected from three locations [Ghor Haditha (GHD), Ghor Safi (GSF), and Ghor Sweimeh (GSW)] nearby the Dead Sea region based on *16S rDNA* sequences and sequences from selected bacteria reference isolates (GenBank accession number for the collected isolates and reference strains are given in Supplementary Table S5). Bootstrap values (>50) are represented by numbers at the nodes based on 10000 replications.

Out of the 74 isolates, 62 were successful in producing the crossponding ∼1500 bp amplification products and their DNA sequencing data were of high quality that enabled their analysis using the blastn tool using the non-redundant (nr) and microbes’ databases (Supplementary Table S4). The majority of the isolates (38 out of 62) belonged to the phylum Firmicutes, class Bacilli and were distributed as follows: 10 isolates belonged to the genus *Bacillus*, nine isolates belonged to genus *Oceanobacilllus*, five belonged to the genus *Salinococcus* and four belonged to genus *Halobacillus*. On the other hand, 18 isolates belonged to the phylum Proteobacteria, 17 of which belonged to class Gammaproteobacteria; with 14 isolates identified as genus *Halomonas*, two as genus *Pseudomonas* and one as genus *Psychrobacter.* A single isolate was identified as genus *Agrobacterium* belonging to class Alphaproteobacteria. Six isolates belonged to phylum Actinobacteria, class Actionobacteria. Interestingly, some endophytic isolates shared a high sequence similarity with rhizospheric isolates from the same collected sample in the same site, e.g., GSF-E-11 with GSF-R-11 from Ghor Safi location (Supplementary Table S4). Based on the collection site, all endophytic and the majority of rhizospheric isolates from Ghor Sweimeh belonged to family Bacillaceae, class Bacilli. Whereas clear diversity was found among isolates obtained from Ghor Safi location (Supplementary Table S4). Phylogenetic analysis with *16S rDNA* sequences of all isolates with reference sequences from related bacteria species confirmed the identification of the tested isolates and grouping at the genus level was observed (Figure 1). Furthermore, four clear clusters representing each identified taxonomical group were identified where Firmicutes was the largest, and Actinobacteria was the smallest. The accession numbers of all identified isolates and reference bacteria sequences are given in Supplementary Table S5.

### *In vitro* Plant Growth Promoting Assays

Based on the phylogenetic analysis and the halotolerance assay results, 35 strains were selected for the *in vitro* growth promoting assays. The selection preference was based on removing redundant samples identified in blastn and phylogenetic analysis and the ability to tolerate high salinity levels in the halotolerance assay. These selected strains were tested for their ability to produce IAA, siderophores, nitrogen fixation, phosphate solubilization and ACC deaminase activity. A reference strain, *A. lipoferum* (ATCC^®^ 29707^TM^), was used as positive control in all assays except in ACC deaminase assay where it was used as negative control. The results of *in vitro* growth promoting assays in all strains are given in Supplementary Table S6. The data of nine selected strains that showed multiple PGP traits (three isolates from each location) plus *A. lipoferum* are presented in Table 1.

**TABLE 1:** Plant growth promoting traits of nine selected strains associated with durum wheat in this study (+ indicates positive; − indicates negative).

	***In vitro* growth promoting assay**				
**Isolate**	**Nitrogen fixation**	**Phosphate mobilization**	**Siderophore production**	**IAA production (μg/ml)**	**ACC deaminase activity**
GHD-E-6	−	−	+	14.76	−
GHD-E-12	+	−	+	9.09	−
GHD-R-3	+	−	+	14.9	−
GSF-E-8	−	−	+	10.69	−
GSF-E-10	−	+	−	13.53	−
GSF-E-11	−	−	−	6.28	+
GSW-E-5	+	+	+	22.41	−
GSW-E-6	+	−	+	18.28	+
GSW-E-7	+	+	+	13.63	−
*A. lipoferum*	+	+	+	27.55	−

#### ACC Deaminase Activity

**FIGURE 2:**
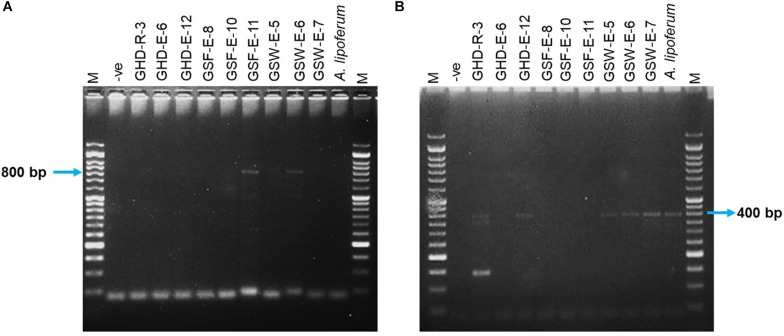
PCR amplification of DNA fragments in *nifH* and *acdS* genes in 10 selected isolates and *Azospirillum lipoferum*. **(A)** The expected DNA fragment of *acdS* is ∼800 bp. **(B)** The expected DNA fragment of *nifH* is ∼390 bp. M: 50 bp DNA ladder (GeneDireX, South Koera); –ve: no bacteria gDNA.

The activity of ACC deaminase in the selected strains was assessed based on their ability to utilize ACC as a sole nitrogen source and were confirmed by PCR amplification of a DNA fragment in the *acdS* gene. Only two endophytic strains (GSF-E-11 and GSW-E-6) were able to degrade ACC and used it as a sole nitrogen source in the media (Table 1 and Supplementary Table S6). Both strains belonged to the Bacilli class with GSF-E-11 closely related to *O. iheyensis*, and GSW-E-6 closely related to *B. subtilis* (Figure 1 and Supplementary Table S4). The PCR amplification of ∼800 bp fragment in the *acdS* gene in both strains confirmed the presence of n *acdS* gene and their ability to degraded the ACC in the media (Figure 2A).

#### Nitrogen Fixation

The ability of the selected strains to fix nitrogen was assessed by the formation of a pellicle in semi-solid NFb media and confirmed by PCR amplification of a 390 bp DNA fragment in the *nifH* gene. Five out of 35 strains were able to form growth pellicles in semi-solid NFb with clear blue coloration in solid NFb plates (Table 1 and Supplementary Table S6). Among the five positive isolates, two (GHD-R-3 and GHD-E-12) were found to be closely related to *H. campaniensis* and *Gracilibacillus timonensis*, respectively; while GSW-E-5, GSW-E-6, and GSW-E-7 were closely related to *B. subtilus* and *B. licheniformis*, respectively (Figure 1 and Supplementary Table S4). PCR amplification of ∼390 bp of *nifH* gene fragment confirmed its presence in the five positive strains as compared to *A. lipoferum* as positive control (Figure 2B).

#### Phosphate Solubilization and Siderophore Production

**FIGURE 3:**
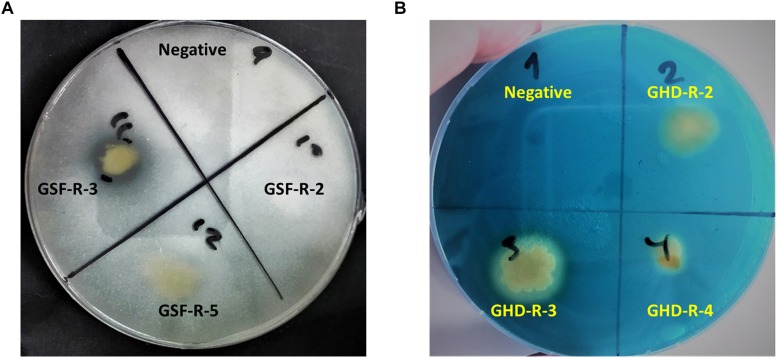
**(A)** Phosphate-solubilizing activity **(B)** and siderophore production in selected halotolerant bacterial strains associated with durum wheat in this study. Negative indicates no bacteria inoculation.

The ability of selected strains to solubilize inorganic phosphate from the media was tested. Among the 35 strains, five were able to solubilize phosphate and formed clear zones on modified Pikovskaya agar plates (Supplementary Table S6). The best strain in solubilizing phosphate was GSF-R-3, which was closely related to *H. titanicae* (Figure 3A).

The production of siderophore was also examined using CAS-blue agar assay; 17 out of 35 strains were able to form halo zones indicting their ability to produce siderophore (Figure 3B, Table 1, and Supplementary Table S6). Strains from Ghor Sweimeh, which belonged to the Bacilli class, were considered good siderophoregenic bacteria (Supplementary Table S6).

#### IAA Production

Compared to *A. lipoferum* strain, GSW-E-5 and GSW-E-6 strains produced the highest levels of IAA (Table 1 and Supplementary Table S6). These two strains were shown to be closely related to *B. subtilis* (Figure 1 and Supplementary Table S4). Only 10 strains were able to produce levels of IAA above 10 μg/ml, while 15 produced levels less than 5 μg/ml. Strains from Ghor Sweimeh, which belonged to the Bacilli class, were considered good IAA producers (six out of seven strains produced IAA levels higher than 12 μg/ml) when compared with strains from other locations (Supplementary Table S6).

#### Antifungal Activity of PGPR Against *F. culmorum*

**FIGURE 4:**
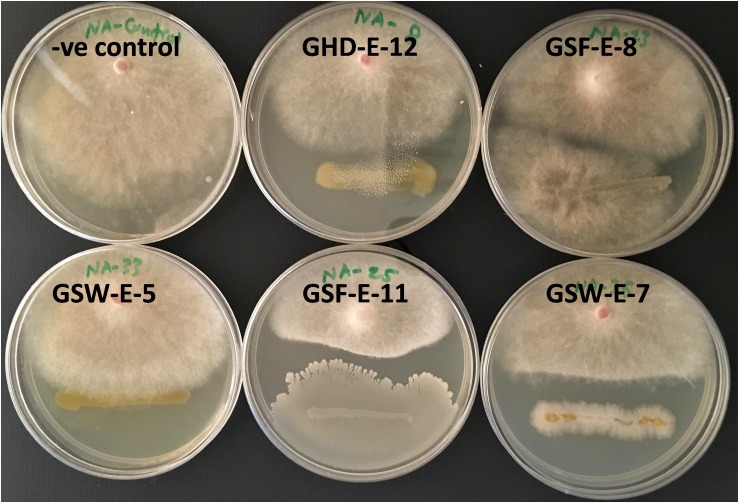
Antifungal effect of halotolerant bacterial strains against *F. culumorum* in a dual culture assay.

Four endophytic bacterial strains, GHD-E-12, GSF-E-11, GSW-E-6 and GSW-E-7, showed antifungal activity against *F. culmorum* restricting mycelial growth in a dual culture plate assay (Figure 4). No antifungal effect against *F. culmorum* was observed in the rest of tested isolates beside *A. lipoferum*.

### Plant Growth Promoting Ability in Durum Wheat Under Stress Conditions

Based on the *in vitro* plant growth promoting assays, nine strains (three per location) with distinguished plant growth promoting features were selected to study their effects on the growth of durum wheat under different stress conditions (Table 1).

#### Salinity Tolerance

The nine selected strains were used to study their effects on the growth of two durum wheat genotypes under different levels of salinity. The combined ANOVA for this experiment showed a high significant (*p* < 0.001) differences for all tested traits among bacteria, salt level, genotype and their interactions (Supplementary Table S7). Salinity levels above 80 mM resulted in significant reduction of germination percentage in inoculated seeds of both tested genotypes (Supplementary Table S8). On the other hand, Line 5004 (*NAX2*) seeds showed significantly higher germination percentages at high NaCl levels (>80 mM) when compared with Tamaroi, the salt sensitive genotype. At salinity level of 200 mM, inoculated seeds with six bacterial strains produced significantly higher germination percentages for each tested genotype, while three strains (GSF-E-8, GSF-E-10, and GSF-E-11) showed no effect or had lower mean values (Supplementary Table S8). For instance, Tamaroi seeds inoculated with GSW-E-6 and GSW-E-7 strains produced mean values of 83.3 and 81.7% for germination percentages, respectively, which were significantly higher when compared with 53.3% in non-inoculated seeds. Whereas, Line 5004 (*NAX2*) inoculated with the same strains produced 100 and 96.7% germination percentages, respectively, which were significantly higher when compared with 66.7% in non-inoculated seeds. Increasing salinity levels up to 200 mM NaCl resulted in the reduction of roots number in all treatments with clear variation among tested strains and wheat genotypes (Supplementary Table S8).

**FIGURE 5:**
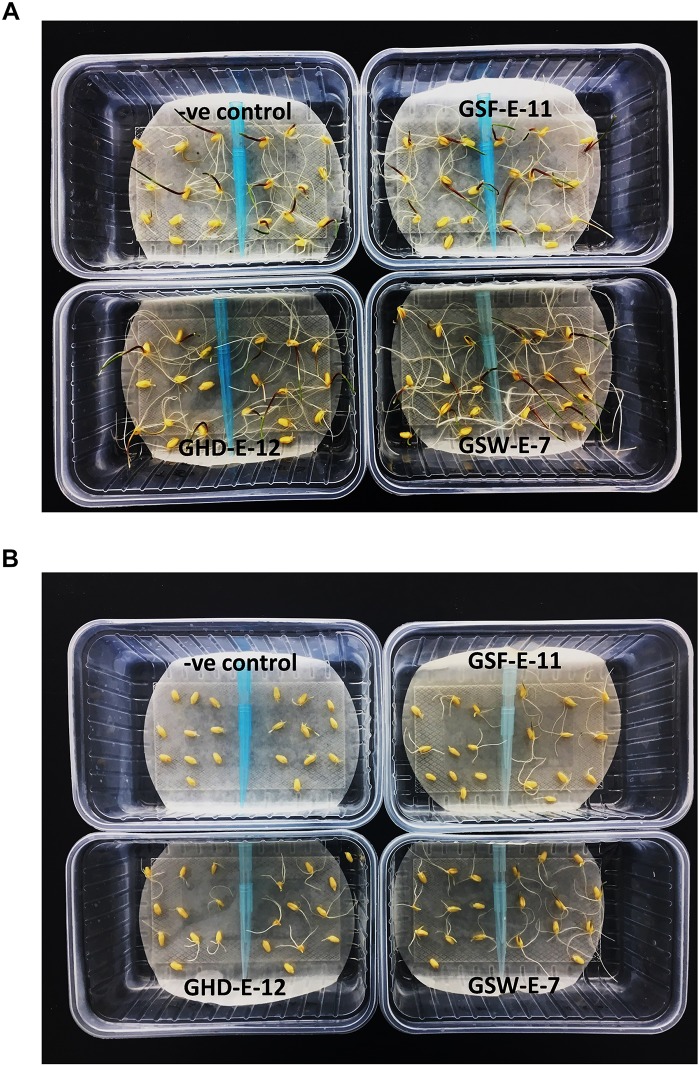
Effect of inoculation with three PGPR bacterial strains, GHD-E-12, GSF-E-11 and GSW-E-7, on the germination of two durum wheat [right: Line 5004 (*NAX2*); left: Tamaroi] after 5 days of culture. **(A)** Non-saline conditions. **(B)** 160 mM NaCl.

Under non-saline conditions, a significant improvement in growth was observed in both genotypes inoculated with some bacterial strains as reflected in higher root length and seedling dry weight mean values (Supplementary Table S8). Only two bacterial strains, GSF-E-8 and GSF-E-10, did not show clear significant effect on growth promotion of both tested genotypes. In Tamaroi, strain GSW-E-6, which is closely related to *B. subtilis* produced the highest mean value for root length (20.67 cm) and was significantly different from non-inoculated seedlings mean value (17.42 cm) (Figure 5A and Supplementary Table S8). No significant differences in Tamaroi root length mean values were observed between GSW-E-6 and GHD-E-12, GSW-E-5 and GSW-E-7 strains under non-saline conditions (Supplementary Table S8). For Line 5004, GSW-E-7 produced the highest mean value under non-saline conditions and was significantly different from the non-inoculated seedlings mean value. For dry weight under non-saline conditions, the lowest mean value was observed with Tamaroi inoculated with GSF-E-8 with no significant difference with non-inoculated seedlings (Supplementary Table S8). In general, seedlings inoculated with seven selected bacterial strains and *A. lipoferum* improved root lengths and root dry weights in both tested genotypes under saline conditions (≥80 mM) when compared with non-inoculated seeds (Figure 5B and Supplementary Table S8). Salinity levels ≥ 80 mM resulted in a significant reduction in root length mean values in non-inoculated seedlings of both genotypes when compared with GHD-R-3, GHD-E-9, GHD-E-12, GSF-E-11, GSW-E-5, GSW-E-6, and GSW-E-7 with higher mean values observed in Line 5004 when compared with Tamaroi (Supplementary Table S8). A significant growth improvement was observed in both tested genotypes inoculated with *A. lipoferum* under saline conditions (Supplementary Table S8).

Root characteristics in terms of root projected area, total root length and root diameter were also analyzed under different NaCl levels (0, 80, and 160 mM) in both wheat genotypes either non-inoculated or inoculated with three selected halotolerant strains (GSF-E-11, GSW-E-6, and GSW-E-7). The combined ANOVA for this experiment showed a high significant (*p* < 0.001) differences for all tested traits among bacteria, salt level, genotype and their interactions except for bacteria × salt level × genotype for root projected area trait (Supplementary Table S7). Under non-saline conditions, the inoculated seedlings with the three strains showed significantly higher mean values for root projected area and total root length in both tested genotypes when compared with non-inoculated seedlings (Table 2).

**TABLE 2:** Effect of inoculation with three halotolerant bacterial strains, GHD-E-12, GSF-E-11 and GSW-E-7, on root characteristics [projected area (mm^2^), length (mm), surface area (mm^2^), and root diameter (mm)] in two durum wheat genotypes under different salinity levels.

		**Projected area**		**Root length**		**Root diameter**	
**Salt level**	**Bacteria**	**Tamaroi**	**Line 5004 (*NAX2*)**	**Tamaroi**	**Line 5004 (*NAX2*)**	**Tamaroi**	**Line 5004 (*NAX2*)**
0 mM	Control	80.94d^*^	80.12d	41.72e	46.22d	0.4905a	0.5101a
	GSF-E-11	109.37a	98.55*bc*	54.96c	60.42b	0.4392a	0.4363a
	GSW-E-6	103.45*ab*	102.14*bc*	62.09b	65.80a	0.4289a	0.4203a
	GSW-E-7	100.86*bc*	95.34c	53.32c	61.36b	0.4138a	0.4177a
80 mM	Control	26.70e	45.56d	10.09f	18.28e	0.6702a	0.5864a
	GSF-E-11	67.53c	85.06*ab*	33.86d	53.95a	0.4459b	0.4377b
	GSW-E-6	62.97c	81.70b	40.77c	44.37b	0.4461b	0.4428b
	GSW-E-7	68.67c	90.98a	39.04c	41.12*bc*	0.4130b	0.4306b
160 mM	Control	2.44d	2.30d	1.66c	0.75c	1.2627b	1.9221a
	GSF-E-11	15.85*bc*	19.28*bc*	10.29b	13.68b	0.5241c	0.4853c
	GSW-E-6	15.81*bc*	33.66a	10.96b	18.02a	0.4915c	0.4872c
	GSW-E-7	13.82c	20.99b	12.34b	18.00a	0.4810c	0.4694c

^*^Means carrying different letters at the same salinity level are significantly different using LSD values at *p ≤* 0.05.

At salinity level of 80 mM, inoculated seedlings produced significantly higher mean values than non-inoculated with a clear difference in salinity tolerance behavior between the two tested genotypes. No significant differences were observed for root projected area between the three strains in Tamaroi genotype, while significant differences in the total root length mean values were observed between GSF-E-11 and GSW-E-6 and GSW-E-7 (Table 2). At salinity level of 160 mM, inoculated seedlings produced significantly higher mean values for both traits when compared with non-inoculated genotypes with clear significant differences between the two tested genotypes (Table 2). For root diameter, no significant differences were observed between treatments under non-saline conditions (Table 2). However, under saline conditions, non-inoculated seedlings produced significantly higher mean values when compared with inoculated seedlings.

To determine the effect of bacterial inoculation on plant growth under salinity stress in hydroponic culture, 1-week old seedlings of Tamaroi and Line 5004 genotypes either non-inoculated or inoculated with GSW-E-7 strain were transferred into a nutrient solution supplemented with different NaCl levels (0, 80, and 160 mM). The combined ANOVA for this experiment showed a high significant (*p* < 0.001) differences for all tested traits among bacteria, salt level, genotype and their interactions except for bacteria × salt level × genotype for root length and shoot fresh weight traits (Supplementary Table S7).

**FIGURE 6:**
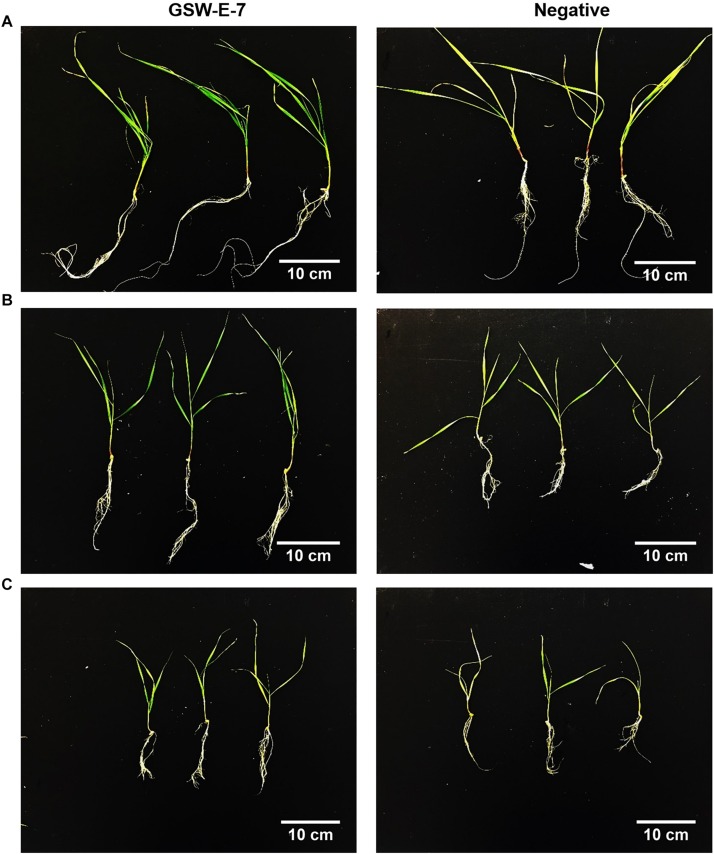
Effect of inoculation with GSW-E-7 strain on growth of durum wheat cv. Tamaroi seedlings after 30 days of culture in hydroponic system. **(A)** 0 mM NaCl; **(B)** 80 mM NaCl; **(C)** 160 mM NaCl. Negative: non-inoculated seeds.

Under non-saline conditions, a significant growth increase was observed in inoculated seedlings of both tested genotypes when compared with non-inoculated seedlings as reflected in higher fresh weight, projected area and total length of both roots and shoots (Figure 6 and Table 3). On the other hand, GSW-E-7 strain inoculated seedlings showed significant root growth at 80 mM salinity level for all measured traits (fresh weight, projected area, and total root length) in both tested genotypes with clear significant differences between the two tested genotypes (Table 3). At salinity level of 160 mM, clear significant differences between inoculated and non-inoculated seedlings were observed for all root growth parameters. However, a clear significant difference was observed between the two tested genotypes only in non-inoculated seedlings, while mean values of inoculated seedlings were non-significant between both genotypes (Table 3).

**TABLE 3:** Effect of inoculation with GSW-E-7 strain on root and shoot characteristics [fresh weight (gm), projected area (mm^2^), and length (mm)] in two durum wheat genotypes under different salinity levels after 3 weeks in a hydroponic culture.

		**Root projected area**		**Root length**		**Root fresh weight**	
**Salt level**	**Bacteria**	**Tamaroi**	**Line 5004**	**Tamaroi**	**Line 5004**	**Tamaroi**	**Line 5004**
0	Control	94.98b^*^	89.29c	152.47c	158.67c	0.0894b	0.0776c
	GSW-E-7	115.32a	117.04a	208.28a	194.87b	0.1067a	0.1144a
80	Control	28.27d	40.21c	49.12d	68.15c	0.0597d	0.0709c
	GSW-E-7	48.05b	56.87a	120.03b	136.63a	0.0926b	0.1045a
160	Control	23.40c	31.83b	30.78c	45.83b	0.0261b	0.0434a
	GSW-E-7	38.94a	42.34a	73.68a	72.89a	0.0419a	0.0442a

		**Shoot projected area**		**Shoot length**		**Shoot fresh weight**	
**Salt level**	**Bacteria**	**Tamaroi**	**Line 5004**	**Tamaroi**	**Line 5004**	**Tamaroi**	**Line 5004**

0	Control	92.68b	99.81b	67.15d	72.75c	0.498b	0.518b
	GSW-E-7	110.48a	112.96a	80.25b	88.52a	0.593a	0.609a
80	Control	74.58b	95.68a	41.15c	57.33a	0.165d	0.2563c
	GSW-E-7	98.41a	95.89a	50.32b	54.28a	0.377b	0.4293a
160	Control	46.73c	59.45*ab*	27.29c	37.51a	0.109a	0.130a
	GSW-E-7	56.24b	64.89a	32.79b	37.32a	0.141a	0.131a

^*^Means carrying different letters at the same salinity level are significantly different using LSD values at *p* ≤ 0.05.

Interestingly, no significant effect of GSW-E-7 strain inoculation on shoot projected area and shoot length in Line 5004 genotype was observed, although a clear effect was observed on inoculated Tamaroi seedling when compared with non-inoculated control (Table 3).

#### Drought Tolerance

**FIGURE 7:**
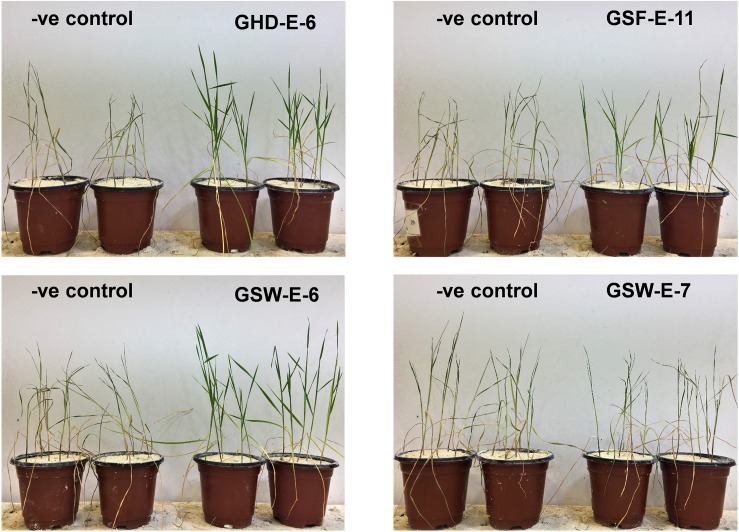
Effect of inoculation with four PGPR bacterial strains, GHD-E-6, GSF-E-11, GSW-E-6 and GSW-E-7, on durum wheat cv. Tamaroi seedling after 10 days of water with-holding. –ve control: non-inoculated seedlings.

Exposing non-inoculated Tamaroi wheat seedling to water withholding for 10 continuous days resulted in pronounced wilting and subsequently the death of all treated seedlings and no recovery was observed after rewatering treatment (Figure 7). Similar responses were obtained for seedling inoculated with other six bacterial strains. On the other hand, the inoculation with GHD-E-6, GSF-E-11, and GSW-E-6 bacterial strains improved water deficit tolerance and survival percentages in Tamaroi seedlings when compared with non-inoculated seedlings (Figure 7).

## Discussion

The Dead Sea is the lowest point on earth (−430 m) and it is located at the tip of the western horn of the fertile crescent where agricultural revolution is believed to have started before 10000 years ago. To the north of the Dead Sea basin, the Jordan valley is considered a core diversity area of wild emmer wheat (*T. turgidum* subsp. *dicoccoides*), the direct progenitor of durum wheat (Weide, 2015). The Dead Sea region climate is considered hot and dry with an annual rainfall level less than 100 mm (Loss and Siddique, 1994) and saline soil conditions is prevalent in many nearby cultivated areas reaching a mean value of EC 14.0 dS/m hindering irrigated agriculture and crop productivity (Ammari et al., 2013).

Targeting such harsh environment where durum wheat had evolved might help in identifying halotolerant PGPR with positive effects on durum wheat growth and development under saline conditions. The isolation of halotolerant PGPR with promoting effect on growth of wheat under saline conditions were reported previously from saline environments (Nabti et al., 2007; Tiwari et al., 2011; Ramadoss et al., 2013) and roots of bread wheat plants (Fischer et al., 2007; Egamberdieva and Kucharova, 2009; Tiwari et al., 2011). Targeting saline soils from northern Algeria resulted in the isolation of the halotolerant *A. brasilense* strain NH that had improved durum wheat growth and development under high salt stress conditions (Nabti et al., 2007). In another study, the *Azotobacter chroococcum* AZ6 strain was isolated from rhizospheric soil surrounding durum wheat plants cultivated in an arid location in Algeria (Silini et al., 2016). Inoculation of durum wheat plants with the AZ6 strain either alone or in combination with osmolytes improved growth under high salt stress. In this study, 74 halotolerant rhizospheric and endophytic bacteria associated with durum wheat were isolated from saline areas in the Ghor region nearby the Dead Sea. Among them, 46 isolates were found to be Gram-positive and 28 isolates were able to tolerate salinity levels up to 20% NaCl. However, to the best of our knowledge, no previous report described the isolation of plant growth promoting endophytic bacteria from durum wheat plants cultivated in hypersaline soils as described in this study. The association between halotolerant PGP rhizospheric and endophytic isolates with a salt sensitive plant such as durum wheat indicates the possibility to mitigate salinity stress and the ability to promote growth under such conditions by indigenous PGPR. This is highly probable knowing that plant samples collected in this study were from saline fields cultivated with the durum wheat cv. *Haurani*, a salt sensitive genotype with salinity threshold for grain yield about 20% at an EC of 7 dS/m (Katerji et al., 2005).

Phylogenetic analysis of 62 halotolerant isolates using *16S rDNA* partial sequences revealed that the obtained isolates were highly diverse at the genus level. The majorities of the isolates were found to belong to the class Bacilli and included eight different genera. Ten isolates were closely related to *Bacillus* spp., five isolates clearly grouped with *B. subtilis* and four isolates were closely related to *B. licheniformis*, all of which are well-known to be beneficial PGPR with growth promotion abilities under different stress conditions that can also induced resistance against different pathogens (Vurukonda et al., 2016; Etesami and Beattie, 2018). Furthermore, nine isolates from the Bacilli class were closely related to *Oceanobacillus* spp., which were previously reported as PGP endophytic bacteria in different plant species (Siddikee et al., 2010; Mapelli et al., 2013) including wheat (Orhan, 2016). On the other hand, 14 isolates were closely related to the genus *Halomonas* (Figure 1), which was reported previously as halotolerant PGPR that can induce tolerance against salinity in wheat plant (Tiwari et al., 2011). Similarly, Upadhyay et al. (2009) reported that the majority of halotolerant bacterial genera identified from rhizospheric soil surrounding bread wheat under saline conditions belonged to the class Bacilli.

The plant growth promoting abilities including nitrogen fixation, inorganic phosphate solubilization, siderophore production, auxin production, ACC deaminase activity, and antifungal effect against *F. culmorum* were assessed in 35 selected isolates. The utilization of halotolerant PGPR with nitrogen fixation ability is considered a good strategy to improve the growth of salt sensitive plants (Etesami and Beattie, 2018). Only five isolates were found to fix nitrogen, three of them (GSW-E-5, GSW-E-6, and GSW-E-7) were closely related to *Bacillus* spp., while GHD-E-12 was related to *Gracilibacillus* spp. and GHD-R-3 was related to *Halomonas* spp. The low incidence of halotolerant isolates with nitrogen fixation in this study is in consistence with Upadhyay et al. (2009), who reported that two out of 24 halotolerant isolates were capable of fixing nitrogen. *Bacillus* spp. with nitrogen fixation ability was identified previously in wheat (Beneduzi et al., 2008) and rice plants (Ji et al., 2014). The halotolerant *B. licheniformis* HSW-16 strain associated with salinity tolerance in wheat was able to fix nitrogen (Singh and Jha, 2016a).

Five halotolerant strains were able to solubilize insoluble phosphate indicating their potential to promote growth of wheat under phosphate-limited conditions as described previously by Upadhyay and Singh (2015). Two PGPR strains closely related to *Halomonas* spp. and *Halobacillus* spp. were able to solubilize phosphate in the presence of salinity and had positive effects on plant growth under such conditions (Desale et al., 2014). Siderophores producing halotolerant PGPR can enhance the uptake of iron by plants (Kloepper et al., 1980) and can also improve plant health by producing antimicrobial compound and depleting metals from rhizosphere affecting pathogen growth and infection (Haas and Défago, 2005). In this study, 17 bacterial strains were found to produce siderophores, which is similar to previous reports where several halotolerant PGPR isolated from wheat were siderophore producers and had growth promoting abilities under saline conditions (Beneduzi et al., 2008; Upadhyay et al., 2009).

The ability of halotolerant PGPR to produce IAA was associated with improved growth of wheat under saline conditions (Nabti et al., 2007; Egamberdieva, 2009; Tiwari et al., 2011). Two strains closely related to *B. subtilis*, GSW-E-5 and GSW-E-6, were able to produce high levels of IAA, which was close to the levels produced by *A. lipoferum* reference strain. Ramadoss et al. (2013) reported the isolation of halotolerant isolates that are related to *Bacillus* spp. with IAA production and growth promoting abilities in wheat. Upadhyay et al. (2012) reported that *B. subtilis* SU47 strain was able to produce IAA and promoted growth of wheat under saline conditions.

Two strains (GSW-E-6 and GSF-E-11) out of 35 were able to utilize ACC as a sole nitrogen source in the media. GSW-E-6 was closely related to *B. subtilis*, while GSF-E-11 was closely related to *O. picturae*. Halotolerant bacteria with ACC deaminase activities closely related to *Bacillus* spp. were reported previously in wheat (Upadhyay et al., 2009; Orhan, 2016; Singh and Jha, 2016a). The ability of PGPR to produce the enzyme ACC deaminase can result in reducing the levels of ethylene hormone by degrading its precursor ACC and subsequently results in promoting plant growth under stress conditions (Glick, 2014).

Recently, several PGPR strains with multiple growth promoting traits improved wheat growth and tolerance against high salinity (Singh and Jha, 2016a, b, 2016c). In this study, three strains closely related to *Bacillus* spp. (GSW-E-5, GSW-E-6, and GSW-E-7) were isolated from the same field site and were found to possess several growth promoting traits such as IAA production, nitrogen fixation and siderophore production that resulted in improving growth of wheat plants under saline conditions. The three strains had pronounced effects on durum wheat seed germination and seedling growth under severe saline conditions. *Bacillus* spp. strains with multiple plant growth promoting traits were found more effective in enhancing wheat growth when compared with single trait strains (Baig et al., 2012). Similarly, strain GSW-E-6, which is closely related to *B. subtilis*, was found to possess multiple plant growth promoting traits including ACC deaminase activity, nitrogen fixation, siderophore and IAA production and had a positive effect on durum wheat germination and seedling growth under severe saline conditions. Furthermore, the same strain improved seedling survival in response to drought by maintaining plant water status and delaying wilting even after 10 days of water withholding. The ability of *Bacillus* strains with ACC deaminase and IAA production activities to improve drought tolerance in different species was reported previously (Vurukonda et al., 2016; Saleem et al., 2018). Strain GSW-E-7, which is closely related to *B. licheniformis* improved substantially wheat growth and development under saline conditions when compared with non-inoculated plants. Similarly, the ACC deaminase and nitrogen fixation bacterium *B. licheniformis* HSW-16 improved wheat growth under saline condition and it possessed other growth promoting traits such as auxin production and phosphate solubilization activity (Singh and Jha, 2016a). Beside salinity tolerance, strain GSW-E-7 has antagonistic effect against an aggressive *F. culmorum* as observed in a dual culture assay. *B. licheniformis* HSW-16 had antagonistic effect against different pathogens including *F. oxysporum* and *F. graminearum* (Singh and Jha, 2016a). Other *Bacillus* spp. were reported to have antifungal effect against *F. culmorum* and induced resistance in durum wheat (Mnasri et al., 2017). The GHD-E-12 strain was found to be closely related to *Gracilibacillus* spp., which was previously reported as an endophytic PGPR in *Arthrocnemum macrostachyum*, a halophyte plant growing in saline agricultural soils (Navarro-Torre et al., 2017).

Few reports described the promoting effect of PGPR on durum wheat growth and development under saline conditions (Nabti et al., 2007, 2010; Silini et al., 2016). In this study, several PGPR strains that belong to different genera were identified as endophytic PGPR in durum wheat, a salt sensitive plant (Gorham, 1990), which was cultivated in saline soils. Introducing the high-affinity K^+^ transporters, *TmHKT1;5-A* (*NAX2*) and *TmHKT7*-*A2* (*NAX1*) from *T. monococcum* into durum wheat, improved high salt tolerance through sodium unloading from xylem tissues reducing its accumulation into toxic levels in the leaf blade (Huang et al., 2006; James et al., 2006). In this study, the inoculation of durum salt-sensitive and salt-tolerant (carries *NAX2*) genotypes with halotolerant PGPR improved germination and seedling growth under high salinity conditions. In the salt sensitive genotype, the inoculation with halotolerant PGPR isolates improved growth under saline conditions, although growth performance was still less than *NAX2* inoculated plants at the same salinity level. This might be related to its weak ability to exclude Na^+^ ions from its root tissue causing reduced growth when compared with the *NAX2* carrying line. Previously, the inoculation of two durum wheat varieties with different levels of salinity tolerance with *A. chroococcum* AZ6 didn’t affect the differential accumulation of the Na^+^ in plant tissues where the salt sensitive Bousselam variety accumulated more Na^+^ in leaves tissues when compared with Waha, a more salt tolerant variety (Silini et al., 2016). In another study, *B. subtilis* strain GB03 induced salt tolerance in bread wheat that was associated with reduced accumulation of Na^+^ in plant tissues accompanied with improved K^+^/Na^+^ ratio (Zhang et al., 2014); the same strain was found to modulate tissue-specific expression of *HKT1* gene in Arabidopsis plant under salt stress conditions and failed to induce salinity tolerance in *athkt1* mutant lines (Zhang et al., 2008). In this study, PGPR strains were successful in improving the growth in a salt sensitive durum wheat genotype lacking *TmHKT1;5-A*, an ortholog to *HKT1;* but whether they have affected spatial accumulation of Na^+^ in plant tissue or modulated the expression of other *HKT* genes in durum wheat, need to be investigated. The substantial increase in roots growth only but not in the shoots under high salt stress conditions in inoculated seedling of *NAX2* carrying line might indicate that ability of the halotolerant PGPR strains to improve tolerance against salinity by other mechanisms beside sodium exclusion that is activated by *TmHKT1;5-A*. Such mechanisms might include exopolysaccharides production, which can bind sodium and decreases its uptake by plants, osmolytes accumulation, ions hemostasis, biofilms formation, and production of phytohormones (Etesami and Beattie, 2018).

## Conclusion

Characterization of several halotolerant PGPR isolated from the rhizosphere of durum wheat plants cultivated in hypersaline environments revealed several growth promoting traits. This was reflected on their ability to alleviate the negative effects of high salinity on durum wheat seed germination and seedling growth. Furthermore, halotolerant PGPR strains with antifungal effect against *F. culmorum* were identified that might have a potential to induce resistance against an aggressive isolate causing crown root rot in durum wheat plants. The identification of halotolerant-multifarious PGPR associated with durum wheat might be used commercially in the future to improve of durum wheat and other crops productivity under saline conditions. However, field testing and studying their efficiency in promoting growth under natural conditions should be considered. Future studies are needed to investigate the mechanisms of induced salinity tolerance by the identified PGPR strains from durum wheat at molecular and physiological levels. Whole genome sequencing of the most promising halotolerant PGPR from this study might also shed the light on and PGP mechanisms associated with induced salinity tolerance in durum wheat.

## Author Contributions

RA conceived most of the research and experimental work, and helped in drafting the manuscript. HK-H designed and conceived the research, helped in data analysis, and drafted the manuscript. JA significantly contributed to the management of the salinity experiments, data analysis, and the interpretation of the results. KA contributed to the antifungal experiments and the interpretation of the results. RA-S contributed to the management of the drought experiments and data analysis.

## Conflict of Interest Statement

The authors declare that the research was conducted in the absence of any commercial or financial relationships that could be construed as a potential conflict of interest.

## Footnotes

1 http://blast.ncbi.nlm.nih.gov

2 http://www.megasoftware.net/
